# Blood biomarkers of amyloid and tau pathologies, brain degeneration, inflammation, and oxidative stress in early‐ and late‐onset Alzheimer's disease

**DOI:** 10.1002/alz.093669

**Published:** 2025-01-09

**Authors:** Xinyi Xia, Qi Qin, Yi Tang

**Affiliations:** ^1^ Department of Neurology & Innovation Center for Neurological Disorders, Xuanwu Hospital, Capital Medical University, National Center for Neurological Disorders, Beijing, Beijing China

## Abstract

**Background:**

Blood biomarkers provide a non‐invasive and accessible means of detection for Alzheimer's disease (AD). Specifically, biomarkers associated with early‐onset AD (EOAD) illuminate the underlying mechanisms and provide valuable guidance for potential therapies. We aim to present blood biomarker evidence in individuals with both EOAD and late‐onset AD (LOAD), as well as in age‐matched healthy controls.

**Methods:**

A total of 95 participants were enrolled in the study. We assessed levels of 18 distinct blood biomarkers, and concurrently examined cerebrospinal fluid amyloid and tau markers, neuropsychological test scores, APOEe4 carrier status, and neuroimaging markers. Group disparities, correlations, and the diagnostic utility of these biomarkers were investigated.

**Results:**

In patients with EOAD, levels of blood IL‐4, IL‐6, and TNF‐a were notably lower when compared to LOAD. AD patients exhibited higher blood levels of p‐tau181, NfL, GFAP, tAOC, and MDA, as well as lower levels of Aß1‐42, SOD, and IL‐12p70. Intricate interactions were observed among these blood biomarkers, with inflammatory markers demonstrating correlations with neuroimaging markers within the AD cohort. Individuals carrying the APOEe4 allele displayed elevated levels of GFAP and IL‐4 in comparison to non‐carriers. Additionally, blood levels of Aß1‐42, p‐tau181, NfL, and GFAP were found to correlate with neuropsychological scores and proved effective in distinguishing AD, with GFAP exhibiting particular relevance in early‐onset cases.

**Conclusion:**

The study demonstrated that individuals with EOAD may experience less inflammatory responses than older patients. Peripheral inflammation was associated with amyloid and tau pathologies, astrogliosis, oxidative stress, and alterations in brain structure and function. Blood Aß1‐42, p‐tau181, NfL, and GFAP showed promise in detecting cognitive decline and AD, with GFAP exhibiting relevance in early‐onset cases.

